# Lichen planopilaris in children: Clinical characteristics, comorbidities, and treatment outcomes in a single-center case series

**DOI:** 10.1016/j.jdcr.2025.09.047

**Published:** 2025-10-27

**Authors:** Carli Needle Lawrence, Anna L. Brinks, Ciara A. Maguire, Jerry Shapiro, Seth J. Orlow, Vikash S. Oza, Kristen I. Lo Sicco

**Affiliations:** The Ronald O. Perelman Department of Dermatology, NYU Grossman School of Medicine, New York, New York

**Keywords:** case series, cicatricial alopecia, inflammation, lichen planopilaris, pediatric

## Introduction

Lichen planopilaris (LPP) is an inflammatory disease of the hair follicle causing frequently irreversible damage and scarring hair loss.^1^ Given the risk of rapid progression and permanence, LPP is considered a “trichologic emergency” warranting timely diagnosis and intervention.^1^ Although LPP is the most common cause of scarring hair loss in adults,^2^ this disease has rarely been described in children. A 2020 systematic review of 985 patients under 18 years of age with lichen planus identified 47 (5%) with LPP.^3^ In a more recent systematic review, there were a total of 20 published pediatric LPP cases.^4^ Prior research on over 1 million patients in a New York City health care system estimated the prevalence of LPP to be less than 0.02%, and no cases were identified in individuals under 21 years of age.^5^

Due to the small number of reported pediatric LPP cases, limited information is available regarding the demographic characteristics, clinicopathologic findings, or treatment outcomes for these young patients. While various therapies exist, intralesional and topical corticosteroids remain the most commonly used treatments for pediatric LPP.^6^ In refractory cases, patients may be prescribed systemic therapies including oral anti-inflammatory medications and Janus kinase (JAK) inhibitors. However, given the limited data regarding pediatric scarring alopecia and effective treatment regimens, selection of therapies is often challenging. Therefore, we performed a retrospective study of 9 children with LPP (7 with confirmatory histopathology) to characterize the clinical presentation, comorbidities, prescribing patterns, and treatment outcomes of LPP in this patient population. This is the largest case series of pediatric LPP to date, with prior series incorporating a maximum of 4 patients.^7^^,^^8^

## Methods

We conducted an Institutional Review Board (IRB)-approved, single-center, retrospective chart review of pediatric patients aged 18 years or younger who presented to NYU Dermatology for LPP over a 15-year period between January 1, 2009, and October 1, 2024. Charts were reviewed for patient demographics, medical comorbidities, age at onset of hair loss and diagnosis of LPP, patient-reported scalp symptoms and psychosocial impacts, trichoscopic features, inflammation severity, treatments, and clinical outcomes. Severity of inflammation was determined via physician assessment, synthesizing dermatoscopic evidence of perifollicular erythema and scale, including thickness of scale and extent of scalp involvement. Data was collected and stored in REDCap.

## Results

A total of 9 children met inclusion criteria (7 male, 7 non-Hispanic White, 2 Hispanic) (Table I). The average follow-up time with NYU Dermatology was 4.7 years. All patients had LPP; concomitant diagnoses included seborrheic dermatitis (*n* = 4), androgenetic alopecia (*n* = 2), and telogen effluvium (*n* = 1). One patient was obese (BMI >30), while 2 had an inflammatory skin disorder (psoriasis, pityriasis rubra pilaris, hidradenitis suppurativa). Of note, the child with psoriasis was on methotrexate (titrated up to 15 mg weekly) for about 2 years prior to the development of LPP. The patient with a history of hidradenitis suppurativa was not on any systemic/biologic therapy for this condition. Three patients had an atopic condition, including atopic dermatitis (*n* = 1), asthma (*n* = 1), and food allergies (*n* = 3); atopic diagnoses overlapped for some patients. There were no notable laboratory abnormalities, hospitalizations, or nutritional deficiencies observed in any of the patients. Relevant family history included autoimmune disease for 5 patients (inflammatory bowel disease, psoriasis, Hashimoto's thyroiditis, Graves' disease, LPP), metabolic disease for 2 patients (obesity, Gilbert syndrome), atopic conditions for 2 patients (atopic dermatitis, allergic rhinitis), and cardiovascular disease for 1 patient (hypercholesterolemia, hypertension) (Table II). Notably, only 1 patient had a family history of LPP, which was diagnosed in an older brother at 8 years of age.

**Table I:** Baseline demographics, diagnoses, and relevant comorbidities

	Patients with pediatric LPP (*N* = 9)
Age at diagnosis (y)	
Mean (SD)	13.3 (4.0)
Gender	
Male	7 (77.8%)
Female	2 (22.2%)
Race/ethnicity	
Non-Hispanic White	7 (77.8%)
Hispanic or Latino	2 (22.2%)
Black or African American	0 (0.0%)
Asian	0 (0.0%)
Middle Eastern or North African	0 (0.0%)
Unknown	0 (0.0%)
Hair/scalp diagnosis∗	
Lichen planopilaris (LPP)	9 (100.0%)
Seborrheic dermatitis	4 (44.4%)
Androgenetic alopecia (AGA)	2 (22.2%)
Telogen effluvium (TE)	1 (11.1%)
Obesity (BMI>30)	
Yes	1 (11.1%)
No	8 (88.9%)
Autoimmune/autoinflammatory comorbidities	
Psoriasis	1 (11.1%)
Pityriasis rubra pilaris	1 (11.1%)
Hidradenitis suppurativa	1 (11.1%)
None	8 (88.9%)
Atopic conditions∗	
Atopic dermatitis	1 (11.1%)
Asthma	1 (11.1%)
Food allergies	3 (33.3%)
None	6 (66.7%)
Average follow-up time (y)	
Mean (SD)	4.7 (6.2)
Age at onset of hair loss (y)	
Mean (SD)	13.3 (4.5)
Duration of hair loss at initial visit (y)	
Mean (SD)	1.8 (20.0)

*BMI*, Body mass index.

∗ Several patients had multiple diagnoses. Thus, the sum of the percents exceeds 100%.

The mean age at onset of hair loss was 11.8 years, the mean age at LPP diagnosis was 13.3 years, and the mean duration of hair loss at initial visit was 1.8 years. Upon initial presentation to NYU Dermatology for scarring alopecia, 5 patients reported scalp itch, 1 reported scalp pain, and none reported scalp burning. LPP was located at the vertex scalp in the majority of patients. Dermatoscopic findings revealed perifollicular scale in 8 patients, perifollicular erythema in 4 patients, and interfollicular erythema in 3 patients. The severity of inflammation was documented as mild in 4 patients, moderate in 1 patient, and severe in 1 patient. Seven patients had a scalp biopsy to confirm the LPP diagnosis. Interestingly, patients with more severe clinical exam findings generally had more pronounced perifollicular fibrosis, lymphocytic and histiocytic inflammation, and follicular dropout on dermatopathology. One patient had nail plate thinning; no patients experienced eyebrow loss, eyelash loss, facial papules, or mucocutaneous lichen planus lesions.

Treatment most commonly consisted of topical corticosteroids (*n* = 7), corticosteroid injections (*n* = 6), hydroxychloroquine (*n* = 5), doxycycline (*n* = 4), TCM solution (tacrolimus 0.3%, clobetasol 0.05%, and minoxidil 5% in a non-propylene glycol vehicle) (*n* = 4), low-dose oral minoxidil (LDOM, 1.25-5 mg daily) (*n* = 4), topical JAK inhibitor (tofacitinib 2% cream [*n* = 1] and ruxolitinib 1.5% cream [*n* = 2]), ketoconazole 2% shampoo (*n* = 3), and topical minoxidil 5% solution/foam (*n* = 2). LDOM (1.25 mg daily) had to be discontinued due to intolerable hypertrichosis in 1 patient, while doxycycline (100 mg BID) had to be discontinued due to rash (*n* = 1), oral fungal infection (*n* = 1), gastrointestinal upset (*n* = 2), headaches (*n* = 1), or aminotransferase elevation (*n* = 1) in 4 total patients. Medications were otherwise well tolerated.

Ultimately, between initial visit and last follow-up visit, LPP improved in 3 patients (resolution of scalp symptoms, no further progression of hair loss, quiescence of inflammatory activity), stabilized in 4 patients (no further progression of hair loss, quiescence of inflammatory activity), and worsened in 1 patient (persistent scalp symptoms, further progression of hair loss, continued inflammatory activity), per dermatologist assessment. There was limited documentation regarding the negative impacts of LPP on psychosocial functioning; however, 1 patient did report embarrassment in public and impairment in daily activities. Similarly, 1 patient reported use of a cranial prosthesis to conceal their alopecia. Study limitations include the lack of racial diversity, small sample size, and associated inability to achieve a high level of statistical power.

## Discussion

Comparing our results to the largest dataset of pediatric LPP to date, we also identified a male preponderance (78% in our study versus 60% in Papierzewska et al).^4^ Interestingly, LPP predominates in females aged 40 to 60 years among adults, although the disease appears to present more commonly in males among children. Furthermore, we similarly reported LPP comorbidities such as atopic dermatitis (11% in our study versus 10% in Papierzewska et al) and psoriasis (11% versus 5%). Papierzewska et al identified additional rheumatological, developmental, and endocrine abnormalities that were not seen in our cohort.^4^ Trichoscopic findings were similar with perifollicular scale in 89% (versus 83%) and perifollicular erythema in 44% (versus 33%); of note, trichoscopic findings were only described for 6 out of 20 patients in the systematic review.

The mean duration of hair loss cited was 16 months (1-60 months); in our study, it was 21.6 months (3-60 months). The wide range in hair loss duration may suggest a delay in diagnosis for some children, potentially due to a lack of scalp burning/pain and the rarity of this disease in children. The presence of scalp itch and scale may initially be attributed to more common etiologies such as seborrheic dermatitis or tinea capitis. In fact, one child completed two 6-week courses of oral fluconazole for a presumed fungal infection prior to receiving an LPP diagnosis. Importantly, the differential diagnosis for LPP in children may include infectious etiologies (tinea capitis), inflammatory dermatoses (seborrheic dermatitis, pityriasis amiantacea), non-scarring alopecias (alopecia areata), and other scarring alopecias (discoid lupus erythematous [DLE], central centrifugal cicatricial alopecia [CCCA], folliculitis decalvans, aplasia cutis, and keratosis follicularis spinulosa decalvans). Sentinel features of LPP in children include perifollicular scale and erythema, loss of follicular ostia, atrophy, and follicular hyperkeratotic papules, located most commonly at the vertex.^4^

LPP is a lymphocyte-mediated alopecia presumed to have an autoimmune pathogenesis and may therefore be associated with other autoimmune conditions.^9^ Our findings corroborate this association, as nearly one-third of our patients had a concomitant autoinflammatory condition. It is possible that LPP, psoriasis, hidradenitis suppurativa, atopic dermatitis, and other autoinflammatory conditions share a related pathogenesis involving dysregulated T cell pathways with resultant inflammation.^10^ This also suggests that patients with a concurrent chronic inflammatory dermatosis, especially those who are not on biologic therapy, may have an inflammatory predisposition for cicatricial alopecia development.

In terms of management, topical corticosteroids were utilized most frequently (78% versus 80%). Interestingly, intralesional corticosteroids were used much more commonly in our cohort (67% versus 15%), possibly related to the older average patient age (13 years versus 11 years). Systemic corticosteroids were not utilized in any of our patients but were used in a quarter of patients included in the systematic review. Several other well-tolerated systemic treatments were much more commonly employed in our cohort: hydroxychloroquine in 56% (versus 5%) to suppress inflammation, and LDOM in 44% (versus 5%) to increase background hair density on the scalp. Doxycycline was used in 44% of our cohort compared to minocycline/doxycycline in one case each from the systematic review. The relatively high rate of doxycycline discontinuation in our cohort underscores the importance of submicrobial/anti-inflammatory doxycycline dosing at 20 mg BID as opposed to 100 mg BID.^11^ In adults, low-dose doxycycline has demonstrated comparable efficacy in targeting inflammation, while decreasing the risk of negative impacts on body flora, antibiotic resistance, and adverse effects, hopefully increasing compliance with therapy.^11^^,^^12^

We also highlight the dearth of information on the psychosocial impact of this disease in children; documentation related to psychosocial effects was only present for 1 patient in our study and no patients in the systematic review. Notably, 1 patient had a history of depression, although the extent to which this was related to their LPP diagnosis remains unclear. Currently, there are no studies focused on the psychological impact of scarring alopecia in children, although pediatric alopecia areata has been associated with a higher risk of anxiety, depression, and reduced self-esteem.^13^ In the adult population, LPP has been reported to have a significant negative impact on quality of life.^14^ It is critical to utilize a holistic management approach in these patients, especially given the lack of available curative therapies.

## Conclusion

Our study represents the largest single-center case series of pediatric LPP and adds valuable insights to the limited existing literature on this rare and potentially devastating condition in children. With a mean clinical follow-up period of almost 5 years, our study found that pediatric LPP may present with distinctive clinical patterns, including a male predominance and vertex scalp involvement, and may be associated with comorbid autoimmune and atopic conditions. Delayed diagnosis also remains a concern; interestingly, the average time from disease onset to diagnosis was 21.6 months for our pediatric cohort, as compared to 33.9 months in adults with LPP.^15^ This considerable diagnostic delay, even among children, may be due to the nonspecific symptoms and misattribution to more common pediatric scalp disorders such as seborrheic dermatitis, tinea capitis, or alopecia areata. Importantly, LPP should never be overlooked as a potential diagnosis, even in younger children. Topical and intralesional corticosteroids remain foundational treatments; however, this study also highlights the utility and tolerability of systemic therapies such as hydroxychloroquine and LDOM. The frequent intolerance of high-dose doxycycline further supports a role for low-dose anti-inflammatory regimens in this population. While our cohort was limited by small sample size and demographic homogeneity, the data underscore the need for greater awareness of pediatric LPP, earlier recognition of hallmark trichoscopic features, and further research into optimal age-appropriate therapies.

**Table II:** Summary of 9 pediatric patients with LPP including demographics, comorbidities, scalp symptoms, trichoscopic features, and disease management

Age/sex	Race/ethnicity	Relevant family Hx	Comorbidities	Scalp Sx	Location on scalp	Trichoscopic findings	Treatments	Length of F/u	Disease course
7/M	Non-Hispanic White	Father: IBD	Seborrheic dermatitis	Itch	Vertex	Thin perifollicular scale, perifollicular and interfollicular erythema	Ketoconazole 2% shampoo TIW, clobetasol 0.05% solution BID, HCQ 200 mg daily, two 6-wk fluconazole courses	4 y	Stabilized
9/M	Non-Hispanic White	Grandfather and first cousin: psoriasis; Mother: allergic rhinitis	Seborrheic dermatitis, psoriasis, pityriasis rubra pilaris, food allergies	Itch	Vertex	Thick perifollicular scale	Topical steroids (regimen ND), lamisil (oral antifungal) 125 mg daily, topical tacrolimus 0.1% ointment BID, tar shampoo	20 y	Stabilized
11/M	Non-Hispanic White	Mother: high cholesterol; Father: HTN; Maternal Uncle: heart disease; Paternal Uncle: HTN, obesity, Hashimoto’s thyroiditis	Seborrheic dermatitis	None	Vertex	Thin perifollicular scale	Ketoconazole 2% shampoo TIW, HCQ 200 mg daily (tapered to TIW)	2.5 y	Improved
12/M	Non-Hispanic White	Older brother: LPP, dx age 8 y	None	Itch	Vertex	Perifollicular erythema, interfollicular erythema	LDOM 2.5 mg daily, IL-TAC monthly, topical steroids (regimen ND), HCQ 200 mg daily, topical tofacitinib 2% cream BID	4 mo	ND
13/M	Hispanic	Mother: AD	Ectodermal dysplasia (subtype not specified), AD, asthma, food allergies	Itch	Mid-scalp and vertex	Thin perifollicular scale	LDOM 1.25 mg daily, topical minoxidil 5% solution daily, IL-TAC monthly, topical clobetasol 0.05% solution, topical tacrolimus 0.3% in CC, doxycycline 100 mg BID, topical ruxolitinib 1.5% daily, tea tree oil, Aminoter Reparage serum	2.9 y	Stabilized
16/M	Non-Hispanic White	Father: Gilbert syndrome	Seborrheic dermatitis, AGA, obesity, depression	None	ND	Thin perifollicular scale, perifollicular erythema, interfollicular erythema	LDOM 5 mg daily, ketoconazole 2% shampoo TIW, dutasteride 0.5 mg daily, IL-TAC monthly, topical clobetasol 0.05% solution, TCM daily, doxycycline 100 mg BID	1 y	Improved
18/M	Non-Hispanic White	None	Hidradenitis suppurativa	Itch, pain	Vertex	Perifollicular scale, perifollicular erythema	IL-TAC (regimen ND), topical clobetasol 0.05% solution daily, HCQ 200 mg BID (tapered down), topical clindamycin PRN	10.6 y	Stabilized
19/F	Non-Hispanic White	None	None	None	Vertex	Perifollicular scale	Oral finasteride 2.5 mg daily, IL-TAC monthly, TCM daily, doxycycline 100 mg BID (tapered down), HCQ 200 mg BID, naltrexone 3 mg daily, pioglitazone 15 mg daily, oral tofacitinib 10 mg BID, topical ruxolitinib 1.5% BID, topical metformin 10% BID in lipo base, metformin 500 mg BID, Excimer laser twice weekly	3 mo	Worsened
19/F	Hispanic	Mother: Graves' disease	AGA, TE, food allergies	None	Vertex	Perifollicular scale	LDOM 2.5 mg daily, topical minoxidil 5% foam daily, IL-TAC monthly, topical clobetasol 0.05% solution daily, TCM BID, doxycycline 100 mg BID	1 y	Improved

*AD*, Atopic dermatitis; *AGA*, androgenetic alopecia; *CC*, cetaphil cleanser; *HCQ*, oral hydroxychloroquine; *HTN*, hypertension; *IBD*, inflammatory bowel disease; *IL-TAC*, intralesional triamcinolone; *LDOM*, low-dose oral minoxidil; *ND*, not documented; *TCM*, topical compounded tacrolimus 0.3%, clobetasol 0.05%, and minoxidil 5% solution; *TE*, telogen effluvium.

## Conflicts of interest

None disclosed.
